# Brain Aging in the Oldest-Old

**DOI:** 10.1155/2010/358531

**Published:** 2010-07-25

**Authors:** A. von Gunten, K. Ebbing, A. Imhof, P. Giannakopoulos, E. Kövari

**Affiliations:** ^1^Service Universitaire de Psychiatrie de l'Age Avancé (SUPAA), Department of Psychiatry of CHUV, University of Lausanne, Route du Mont, 1008 Prilly, Switzerland; ^2^Department of Psychiatry, HUG, Belle-Idée, University of Geneva School of Medicine, 1225 Geneva, Switzerland; ^3^Unité de Psychopathologie Morphologique, Department of Psychiatry of HUG, 1225 Genève, Switzerland

## Abstract

Nonagenarians and centenarians represent a quickly growing age group worldwide. In parallel, the prevalence of dementia increases substantially, but how to define dementia in this oldest-old age segment remains unclear. Although the idea that the risk of Alzheimer's disease (AD) decreases after age 90 has now been questioned, the oldest-old still represent a population relatively resistant to degenerative brain processes. Brain aging is characterised by the formation of neurofibrillary tangles (NFTs) and senile plaques (SPs) as well as neuronal and synaptic loss in both cognitively intact individuals and patients with AD. In nondemented cases NFTs are usually restricted to the hippocampal formation, whereas the progressive involvement of the association areas in the temporal neocortex parallels the development of overt clinical signs of dementia. In contrast, there is little correlation between the quantitative distribution of SP and AD severity. The pattern of lesion distribution and neuronal loss changes in extreme aging relative to the younger-old. In contrast to younger cases where dementia is mainly related to severe NFT formation within adjacent components of the medial and inferior aspects of the temporal cortex, oldest-old individuals display a preferential involvement of the anterior part of the CA1 field of the hippocampus whereas the inferior temporal and frontal association areas are relatively spared. This pattern suggests that both the extent of NFT development in the hippocampus as well as a displacement of subregional NFT distribution within the Cornu ammonis (CA) fields may be key determinants of dementia in the very old. Cortical association areas are relatively preserved. The progression of NFT formation across increasing cognitive impairment was significantly slower in nonagenarians and centenarians compared to younger cases in the CA1 field and entorhinal cortex. The total amount of amyloid and the neuronal loss in these regions were also significantly lower than those reported in younger AD cases. Overall, there is evidence that pathological substrates of cognitive deterioration in the oldest-old are different from those observed in the younger-old. Microvascular parameters such as mean capillary diameters may be key factors to consider for the prediction of cognitive decline in the oldest-old. Neuropathological particularities of the oldest-old may be related to “longevity-enabling” genes although little or nothing is known in this promising field of future research.

## 1. Introduction

The rapid growth of the world population's oldest-old age segment has prompted awareness of age-related diseases including dementia as well as considerable interest in the study of the aging human brain. By 2020, people older than 60 years will account for more than 20% of the total population with those individuals reaching very old ages corresponding to the fastest growing age group worldwide. Rare at the beginning of the 20th century in Switzerland with about 650 people aged 90 or more, a steady increase occurred with a total of 2000 oldest-old persons by 1945. Near exponential growth of this oldest-old group occurred after 1945 with the total number of oldest-old citizens amounting to 47000 by 2001, that is, a multiplication of 25 in 56 years [1]. The number of centenarians was multiplied by 66 since 1950. The Swiss Federal Office for Statistics predicts between 90000 and 100000 persons over 90 years by 2040 and between 110000 and 146000 by 2060 with life expectancy in women possibly reaching 90 years. Similarly, the number of centenarians increases steadily in France, the USA, New Zealand, Japan (for review see [2–4]), and also in the African American community notwithstanding a lower life expectancy at birth [5].

Our ancestral fascination for extreme aging and the steady increase of the number of centenarians worldwide prodded the research community to look into psychobiological particularities of the “longevity outliers”. The known socio demographic predictors of mortality such as smoking and obesity are less important in this age group [6–11]. Centenarians may be less prone to oxidative stress and have better nutritional status, immunologic profile, endocrinologic and metabolic characteristics than younger elderly cohorts [12, 13]. The oldest-old may have on average a better health with a rapid terminal decline relative to those who die earlier [14]. They report greater satisfaction with life and social and family relations and display lower scores for anxiety and depression and better coping abilities compared to younger-old individuals [15]. In the oldest-old, good health is associated with greater intellectual activity, while greater social activity was predicted by extraversion and, interestingly, negative life events [16]. Thus, centenarians may form a select cohort with relatively slow rates of aging and increased resistance to biological and psychological stress.

## 2. Dementia in the Oldest-Old

Life expectancy with or without incapacity has increased markedly: some subjects may remain independent for long periods into older age while others require help and care over extended periods of their lives. In Switzerland, the proportion of persons living in nursing homes increases from only 5% for those below 80 years to 40% for those between 90 and 94 years of age. Dementia clearly contributes to this reality. However, it remains unclear how to define dementia in the frail oldest-old and what it means to be a dementia-free centenarian despite the claim of some researches that up to a quarter of all centenarians may be cognitively intact [17, 18]. Thus, it does not come as a surprise that trustworthy prevalence and incidence data are still scarce in the oldest-old age group. In a community sample of 250000 people, 17 centenarians were traced down and 15 could be examined of whom all had cognitive impairment with a CDR ranging from 1 to 3 [19]. The authors' most conservative estimation of dementia in those 100 years old or more amounted to 88%. Very old age has long been thought to be associated with the highest prevalence of dementia [19–21]. More recent epidemiological and clinical studies in larger cohorts of very old individuals showed prevalence rates which varied widely. This variability suggests, on the one hand, that substantial methodological difficulties remain and, on the other hand, that dementia is not inevitable in very old individuals (for review see [2, 17]) and may even decrease after age 90 [22–24]. However, the pendulum is currently swinging back and preliminary results now indicate that dementia and AD continue to rise also at very high ages with both incidence and prevalence of dementia being highest in the oldest-old [25, 26]. Although further studies with better operational criteria for dementia in the oldest-old are still needed to settle these controversies, epidemiological and clinical studies nevertheless indicate that the oldest-old are likely to be biologically different from the younger-old. The lack of an association between Alzheimer's disease (AD) and ApoE4—a known risk factor for late-onset AD in younger cohorts—in centenarians adds further evidence to this hypothesis [27–30]. The study of oldest-old individuals may permit to define the spectrum and extent of changes in brain morphology that occur with normal brain aging and assess correlations between the neuropathological definition of normal brain aging and the clinical development of dementia [31–33].

## 3. AD-Related Lesion Distribution Patterns in the Normal Elderly

Brain aging is characterized by the formation of neurofibrillary tangles (NFTs) and senile plaques (SPs) as well as neuronal and synaptic loss in both cognitively intact individuals and patients with AD. The first cited diagnostic criteria for AD were intended to aid in the development of uniform procedures by proposing minimally required SP densities as a function of age [34, 35]. These recommendations were not broadly accepted and the Consortium to Establish a Registry for Alzheimer's Disease (CERAD) proposed another set of standardized neuropathological criteria [36]. These semiquantitative criteria were determined as a function of the development of neuritic plaques in three age groups (less than 50, 50 to 75, and over 75). The diagnosis was based on a combination of clinical information and an “age-related plaque score” that reflected the maximal cortical involvement. This combination yielded a tripartite level of diagnostic certainty (i.e., definite, probable, or possible AD). However, the lesion load in the hippocampal formation was not entered into the diagnostic algorithm despite its involvement in the pathogenesis of AD. At that, CERAD criteria have been inspired somewhat unilaterally by the amyloid cascade hypothesis and do not consider NFT densities in the neocortex, even though the latter correlate better with the severity of dementia. In other words, severe SP formation may take place in the neocortex in the presence of only very mild cognitive impairment (for review see [37, 38]). Furthermore, the central pathological hallmark of AD, that is, NFT, is found in subjects with no significant cognitive impairment. Indeed, there is a significant overlap in NFT and neuritic plaques burden between cognitively impaired and cognitively intact individuals [39]. Thus, in 97 nondemented people with a mean age of 84 years, about 40% met at least some level of and 20% strict criteria for neuropathological criteria for AD [40]. Clearly, the clinical significance of NFT is not unequivocal and other pathological predictors are likely to exist of which reduced neuron number appears to be a candidate. Indeed, stereological analyses have revealed age-related decreases in total neuron number of 30% and 50% in the dentate hilus of the hippocampus and subiculum, respectively, between ages 13 and 85. Conversely, no neuronal loss was found in CA1-3 fields and entorhinal cortex during normal aging in contrast to AD [41–43]. In normal aging, there is no additional depletion, as in AD, of neuronal cell bodies in the dentate hilus and subiculum, or a massive reduction in the numbers of pyramidal neurons in the CA1 field and layers II and V of the entorhinal cortex [41–45].

## 4. Clinicopathological Correlations in Typical Alzheimer's Disease

While the definite diagnosis of AD is based on neuropathological criteria, the clinical diagnosis of probable and possible AD in clinical settings is usually made according to the NINCDS-ADRDA criteria [46]. Typical AD is characterized by an insidious onset and a progressively worsening course of episodic memory. The most common initial presentation of AD is that of a progressive amnestic syndrome [47]. Executive, linguistic, visuospatial, and other cognitive deficits are subsequently grafted upon the primary progressive memory impairment with functional deficits and increasing dependency paralleling the course of the cognitive decline. Prospective studies of large cohorts of patients with typical AD have shown a prototypical and predictable clinical course [48] although important atypical variations often occur (for a review [49]). 

In terms of clinicopathological correlations, several lines of evidence indicate that the primary progressive amnestic syndrome so characteristic of the initial stages of typical AD is the consequence of the neuropathological changes in the medial temporal structures, in particular the entorhinal cortex and the hippocampus. Although there is still ongoing controversy as to the exact roles of these structures in cognition, they are likely to be important in encoding new information [50–52]. The initial stages of AD are characterized by NFT spread from the entorhinal cortex to the hippocampus, corresponding to Braak and Braak stages 1 and 2, which precedes the progressive invasion of the allocortex and isocortex [53]. The NFT distribution is not only area-specific but also cell-specific. In the hippocampus, particularly in the CA1 and CA2 regions, pyramidal cells are selectively damaged whereas glutamatergic cells degenerate in the entorhinal cortex presumably interrupting complex neuronal circuits in the medial temporal lobe that are indispensable for encoding new information [54–57]. Besides episodic memory deficits, early impairment of olfactory perception has been described and kindled hope that this observation might allow early and easy detection of AD [58–61]. Besides the early damage of the limbic system, previous clinicopathological studies also revealed strong relationships between the patterns of NFT distribution and cognitive deficits in typical AD cases. For instance, constructional apraxia correlated with NFT densities in Brodmann areas 7 and 18 [62], and other specific correlations were found for associative visual agnosia [63], naming and identification of famous faces [64], and spatial disorientation [65]. In contrast to NFT, SP correlate less well with clinical features and their presence may be associated with no or only minimal intellectual changes in the elderly (for a review see [50–52]). 

## 5. AD-Related Lesion Distribution Patterns in the Oldest-Old

Most of the above studies have not included very old subjects. The question, thus, arises whether or not the pattern of lesion distribution and neuronal loss changes in extreme aging. 

### 5.1. Oldest-Old versus Younger-Old with or without Dementia (cf. also Table 1 for a Schematic Representation)

A recent study using the CERAD criteria for neuropathological diagnosis found a progressive increase of moderate to severe AD-type pathology with age in subjects between 69 and 103 years in those without dementia [66]. In the demented, dementia status is mainly related to severe NFT formation within adjacent components of the medial and inferior aspects of the temporal cortex in younger cases whereas oldest-old individuals with dementia display a preferential involvement of the anterior part of the CA1 field of the hippocampus with relative sparing of the inferior temporal and frontal association areas [44, 67]. The progression of NFT formation across the different CDR groups was significantly slower in nonagenarians and centenarians (from 1 to 17% in the entorhinal cortex and 1.7 to 37% in the CA1 field) compared to recent observations in a series of younger cases (from 4 to 79% in the entorhinal cortex and 3 to 80% in the CA1 field) [68]. The degree of interindividual variability for NFT numbers was, however, quite similar between younger and elderly cohorts [68]. The Oregon brain aging study on neuropathologic aging and cognitive function in healthy oldest-old individuals confirmed this pattern of NFT distribution in the CA1 field [32]. In agreement with previous observations in centenarian brains [44], even cases with moderate dementia display only mild NFT formation in the entorhinal cortex with more than 80% of preserved neurons. This contrasts with the results of several previous studies in younger samples which demonstrated that the entorhinal cortex is more severely affected and involved earlier in the degenerative process than hippocampal regions [38, 69–71]. 

The magnitude of neuronal loss in the entorhinal cortex and CA1 field in older subjects was significantly lower than that reported in younger AD cases [45, 58, 68, 72, 73]. Moreover, the extensive neuronal loss in the hippocampal formation reported in younger AD series [74] appears to be confined to layer II of the entorhinal cortex in nonagenarians and centenarians [44]. In this latter group, the number of layer II entorhinal cortex neurons is thought to decrease by 60% in patients with CDR 0.5 and by 90% in severe AD cases [45]. In the CA1 field, a depletion of 38% to 69% was reported [45, 58, 68, 72, 73]. These data imply that, like AD pathologic changes, neuronal loss is less prominent in the oldest-old even in the presence of AD [44]. In conjunction with the observations of only mild synaptic loss and cerebral amyloid angiopathy in centenarians [75] these findings give additional support to the notion that the occurrence and progression of AD-related pathologic changes are not a sine qua non concomitant of increasing age [32, 44, 76, 77].

Strong relationships between NFT counts and neuron loss in the hippocampal formation and neocortex have been reported and suggest that neuronal loss is NFT-dependent [45]. However, our data reveal a dissociation between the patterns of progression of NFT and neuronal loss in the entorhinal cortex and CA1 field [78]. Non-NFT related mechanisms of neurodegeneration may therefore determine neuronal depletion in the oldest-old age group [44]. These mechanisms remain largely speculative, but recent contributions postulate that apoptosis, oxidative stress and excitotoxic mechanisms play a key role in inducing neuronal death that would predate NFT formation in some regions (for review see [73]). 

Unlike younger cohorts where SP formation does not correlate with neuronal depletion and cognitive status [79–81], both earlier and more recent studies suggest that SP densities in the neocortex are related to the degree of neuronal loss and severe AD in the oldest-old [32, 44]. However, older cases also display significantly lower total amyloid volume in the areas studied compared to that reported in younger series (20 mm^3^ versus 100–800 mm^3^) [82]. 

### 5.2. Demented Oldest-Old versus Nondemented Oldest-Old (cf. also Table 2 for a Schematic Representation)

As mentioned above, the association between AD-type pathology and dementia, in a cohort of 456 subjects between 69 and 103 years was stronger in the younger old persons than in the older old ones [66]. Oldest-old individuals with dementia display a preferential involvement of the anterior part of the CA1 field of the hippocampus with relative sparing of the inferior temporal and frontal association areas [44, 67]. Thus, NFT development in the hippocampus may be the key determinant of dementia in the very old. In line with this view, higher NFT densities were found in the CA1 field of one demented centenarian as compared to eleven cognitively intact centenarians [83]. NFT densities in the anterior CA1 but not in the posterior CA1 field and entorhinal cortex, were significantly different between demented and nondemented very old patients [31, 44, 77]. However, other studies find evidence for more extensive brain involvement in the oldest-old with dementia. In a such study of 19 centenarians including four AD cases, substantial NFT involvement was present not only in the hippocampus but also the entorhinal cortex [84]. 

### 5.3. Clinicopathological Correlations in the Oldest-Old

#### 5.3.1. AD-Pathology

Several cases with minimal AD pathology and preserved cognitive functions [31, 33, 67], so called “supernormal centenarians”, represent a rare phenotype relatively protected from AD pathology and bear witness to successful aging near the upper limit of the human life span. Recent studies attempted to define the cognitive impact of NFT, SP, and neuronal loss in this age group. As mentioned, the strength of the association between overall AD pathology and dementia declined with age [66]. However, not only the global lesion load, but also the lesion distribution may play a role. Thus, AD-related pathology including the assessment of total NFT, neuron numbers, and amyloid volume in entorhinal cortex, CA fields, and dentate gyrus was performed in 12 individuals over 90 years with variable degrees of cognitive decline [85]. Total neuron numbers and volumes of reference were fairly consistent among cases. In fact, the estimates of these variables fall well within the range of previous stereologic assessments in these regions from cognitively intact elderly individuals [45, 58, 68, 72, 74, 82, 86]. As mentioned, even cases with moderate dementia display only mild NFT formation and neuron loss in the entorhinal cortex while the entorhinal cortex is more severely affected and involved earlier in the degenerative process than other hippocampal regions in younger samples [38, 69–71]. Strikingly, correlations between AD pathological hallmarks in the hippocampal formation and clinical status after 90 years were poor [85]. Only a modest percentage of the CDR variability was explained by NFT counts in CA2-3 (18%) and the dentate gyrus (17%). Neither neuron numbers nor total amyloid volumes were significantly related to the CDR score. In spite of the clear neuronal loss observed in cases with moderate to severe dementia, total neuron numbers in the entire sample did not significantly predict cognitive status. Overall, sparing of the entorhinal cortex and CA1 field in the oldest-old relative to younger cohorts suggests that independent morphometric variables may decisively contribute to the cognitive decline in this age group. 

Indeed, neuritic dystrophy [87] may be a further contributor. Neuropil threads are thought to account for 85-90% of cortical tau pathology in normal brain ageing [88] and, thus, they may contribute to cognitive deterioration. However, neuropil thread formation in the hippocampus did not appear to be an independent marker of dementia severity as their length was strongly correlated with NFT numbers at least in the early stages of the degenerative process in the oldest-old [89]. Progression of hippocampal and entorhinal tau burden was associated with dementia status, but this effect disappeared when adjusted for Braak and Braak stages [90].

### 5.4. Other Pathological Changes

Synaptic loss may be an important factor [87]. Recently, significant hypertrophy of the cell bodies, nuclei, and nucleoli in the CA1 neurons was found in elderly nuns with normal cognition but substantial AD-type lesions suggesting that neuronal hypertrophy may constitute an early cellular response to AD or reflect compensatory mechanisms [91]. Structural parameters of the cerebral vasculature such as perivascular collagen deposits, atrophy of endothelium, basement membrane thickening and pericyte degeneration as well as qualitative changes in microvascular structure (such as glomerular loops and twisted capillaries) as described both in the aging brain and in AD offer themselves as still further contributors to cognitive decline (for review see [87, 92–95]). Quantitative analyses of structural parameters such as capillary density, diameters, or length led to controversial data [96–103]. The development of modern design-based stereological techniques allowed for a more accurate assessment of age-related changes in the capillary network [104, 105] and open up this field of study to nonagenarians and centenarians. In 19 very old individuals with various degrees of cognitive impairment, both mean diameters and total capillary numbers, but not total capillary length and capillary morphological parameters, were strongly related to total neuron numbers in the CA1 field and entorhinal cortex [106]. These results suggest a relationship between microvascular changes and AD-related neuronal depletion. Disruption of the balance between energy requirements and cerebral blood supply rendering the brain more vulnerable to oxidative stress damage and ultimately neuronal death may explain this link [93, 107–109]. Mean capillary diameters in the CA1 field and entorhinal cortex explained respectively 19% and 31.1% of the CDR scores, an association that persisted after adjustment for total neuron numbers, NFT numbers, or amyloid volume [106]. Instead of the recruitment of additional capillaries, increased cognitive load may induce differential distribution of flow [110], heterogeneity in blood flow velocity [111], and changes in capillary diameter [112]. 

In the longitudinal Oregon Brain Aging study, NFT and SP densities in neocortical areas were significantly related to cognitive scores [32]. Overt clinical signs of AD in oldest-old individuals appears to require a progressive damage of areas 7, 22, 23 and 24 suggesting a displacement of NFT, such that parietal and cingulate cortex are more affected than is usually the case in AD, whereas superior frontal and inferior temporal association areas are relatively preserved [44, 71].

## 6. Conclusions

The controversy over the continuity versus discontinuity between normal brain aging and dementia goes on. The hypothesis that AD is an ageing-related condition is supported by the nearly ubiquitous presence of AD pathologic changes in the course of brain aging and the exponential increase of AD prevalence after 65 years of age. However, contrasting with this view of the aging process, the study of oldest-old individuals indicates that the occurrence of AD pathology is not a mandatory phenomenon of increasing chronologic age. In particular, the neuropathology of advanced aging strongly suggests that very old individuals with AD display a striking resistance to the neurodegenerative process with only mild neuronal loss in the hippocampal formation. Our hospital-based findings support a dissociation between the clinical expression and traditionally assessed AD pathology. These findings are consistent with the recent neuropathological observations of the New England Centenarian Study [8, 113] and show the limits of the lesional model for explaining the expression of dementia symptoms in this particular age group. Furthermore, they offer a new perspective in the field of clinicopathological correlations by revealing the cognitive impact of decreased capillary diameter, a structural but not lesional parameter of the aging brain. Similarly, we are not to forget changes at the cellular level reflecting both early cell stress to AD or compensatory mechanisms [91]. A further conclusion stemming from the observation of a looser association between AD-pathology and dementia in the oldest-old suggests the necessity to adapt current neuropathological criteria for the diagnosis of AD in this age group [114]. 

The biological background of the increased resistance to AD lesion development after 90 years is still poorly understood. Oldest-old people may show genetic variations that influence basic mechanisms of brain aging resulting in a decreased susceptibility to age-associated diseases [115]. In particular, the oldest-old may lack “disease genes” that predispose to fatal age-related diseases [116], or, alternatively, have so called “longevity-enabling” genes that confer protection against age-related illnesses and constitute future therapeutic targets to delay late-onset dementia [117, 118]. The sparing of CA1 field and entorhinal cortex in the oldest-old may be related to a genetically determined resistance of these neuronal subpopulations in very old people. Two candidate genes predisposing to longevity related to lipoprotein synthesis have been identified and suggest that protection against cardiovascular diseases may be primordial to achieve extreme old age [119–121]. Although the relationship between these vascular “longevity-enabling” genes and AD-related pathology is not clear, the link between preserved cognition and brain capillary diameter points to the need for exploring the genetic determinants of the microvascular system in very old individuals as well as possible links with compensatory mechanisms at the cellular level [91]. The development of structural investigation methods at the microscopic level in combination with differential gene expression studies may provide the future basis for a better understanding of longevity.

## Figures and Tables

**Table 1:** Alzheimer's disease pathology in the cognitively impaired young-old versus oldest-old.

	Cognitively impaired young-old subjects	Cognitively impaired oldest-old subjects
Senile plaques	Higher amyloid load but lower correlation with neurone loss and cognitive status	Lower amyloid load but better correlation with neurone loss and cognitive status

	Early and significant CA2-3 involvement	Invasion of anterior CA1 field
	Early and significant EC involvement	Mild invasion of EC
	Significant inferior temporal and frontal associative cortex involvement with increasing dementia	Relative sparing of inferior temporal and frontal associative cortex
Neurofibrillary tangles	Less parietal and cingulate cortex involvement	More parietal and cingulate cortex involvement
	With advancing dementia, quick invasion of CA1 andspread to adjacent associative cortex	With advancing dementia, lower invasion of CA1 and less spread to associative cortex
	Higher strength of association with dementia	Lower strength of association with dementia

	High interindividual variability	

Neurones	Loss of pyramidal neurones in CA1 and EC	Less neurone loss in CA1 Possibly, relative sparing of EC neurones
	More NFT-related neurone loss	Less NFT-related neurone loss

NFT: neurofibrillary tangles; CA: Cornu Ammonis; EC: entorhinal cortex.

**Table 2:** Alzheimer's disease pathology in the oldest-old with versus without cognitive impairment.

	Cognitively intact oldest-old subjects	Cognitively impaired oldest-old subjects
Senile plaques	Similar lesion load	Similar lesion load
	Overall, poor correlation with clinical status	

	No or little invasion of anterior CA1 field	Invasion of anterior CA1 field
	Similar density in posterior CA1field	Similar density in posterior CA1 field
Neurofibrillary tangles	Similar, mild invasion of EC	Similar, mild invasion of EC (controversial)
	Possibly less neuritic dystrophy	Possibly more neuritic dystrophy
	Overall, poor correlation with clinical status	

Neurones	Similar neurone counts	Similar neurone counts
	Overall, poor correlation with clinical status	

CA: Cornu Ammonis; EC: entorhinal cortex.
